# Diabetic Ketoacidosis as First Presentation of Latent Autoimmune Diabetes in Adult

**DOI:** 10.1155/2015/821397

**Published:** 2015-03-05

**Authors:** Omar Nadhem, Essam Nakhla, Roger D. Smalligan

**Affiliations:** Internal Medicine Department, Texas Tech University Health Sciences Center, Amarillo, TX 79106, USA

## Abstract

A 54-year-old white female with hypothyroidism presented with abdominal pain, nausea, vomiting, and diarrhea. She was found to have diabetic ketoacidosis (DKA) and admitted to our hospital for treatment. Laboratory workup revealed positive antiglutamic acid decarboxylase antibodies and subsequently she was diagnosed with latent onset autoimmune diabetes in adult (LADA). She was successfully treated with insulin with clinical and laboratory improvement. Diagnosis of LADA has been based on three criteria as given by The Immunology of Diabetes Society: (1) adult age of onset (>30 years of age); (2) presence of at least one circulating autoantibody (GADA/ICA/IAA/IA-2); and (3) initial insulin independence for the first six months. The importance of this case is the unlikely presentation of LADA. We believe that more research is needed to determine the exact proportion of LADA patients who first present with DKA, since similar cases have only been seen in case reports. Adult patients who are obese and have high blood sugar may deserve screening for LADA, especially in the presence of other autoimmune diseases. Those patients once diagnosed with LADA need extensive diabetic education including potentially serious events such as diabetic ketoacidosis.

## 1. Introduction

Latent autoimmune diabetes in adult (LADA) is an autoimmune endocrine disorder in which, despite the presence of pancreatic islet antibodies at diagnosis of diabetes, the progression of autoimmune beta cell failure is slow [1, 2]. LADA accounts for 2–12% of all cases of diabetes in adult population [3]. The diagnosis can be made if there is evidence of adult-onset non-insulin-requiring diabetes (>30 years of age), positive circulating autoantibodies, and no need for insulin treatment during several months after diagnosis [3–5]. Patients with LADA usually tend to be younger and leaner [4] and do not present with ketoacidosis at the time of diagnosis, owing to the slow progression of beta cell destruction [6]. Acute hyperglycemic crisis in the form of diabetic ketoacidosis (DKA) or hyperglycemic hyperosmolar state (HHS) is unusual finding [7, 8]. We report a case of an obese patient who presented with gastroenteritis and was found to have diabetic ketoacidosis and subsequently was diagnosed with LADA.

## 2. Case Report

A 54-year-old white female presented with abdominal pain, nausea, vomiting, and diarrhea (five watery bowel movements every day) for 2 days. The patient states that she has been eating a regular diet and denied eating outside the home and did not have fever, and no one in the family had similar symptoms. Past medical history was positive for hypothyroidism and bipolar disorder and family history was negative for diabetes. One year prior to this presentation the patient had routine blood work with fasting blood glucose of 95 mg/dL. The patient reported polyuria and polydipsia for one month prior to this presentation but did not seek any medical attention.

On physical examination her blood pressure was 127/65 mmHg, heart rate 80 beat/min, and temperature 37°C. Her body mass index was 35 kg/m^2^. She was in mild distress and had no thyromegaly, clear lungs, regular heart without murmurs, soft abdomen but with generalized tenderness, and hyperactive bowel sounds throughout. The remainder of the physical exam was normal.

### 2.1. Laboratory Tests

WBC 14.8 × 109/L with neutrophils 89%, hemoglobin 18.2 g/L, and platelets 241 × 109/L. Her chemistry shows sodium 132 mmol/L, potassium 3.9 mmol/L, chloride 99 mmol/L, bicarbonate 12 mmol/L, BUN 5.71 mmol/L, creatinine 53.3 *μ*mol/L, calcium 2.35 mmol/L, phosphorus 1.55 mmol/L, magnesium 1.1 mmol/L, and blood glucose 18.76 mmol/L. Arterial blood gases show PH 7.25, Pco2 3.72 kPa, and Po2 9.58 kPa on room air. Lactic acid was 1 mmol/L, amylase 1.1 *μ*kat/L, and lipase 0.27 *μ*kat/L, and serum ketones were positive. Her hemoglobin A1C was 13.7%. Urinalysis was positive for glucose and ketones.

It was obvious from the labs that the patient had diabetic ketoacidosis (high blood glucose, low bicarbonate, and positive ketones) with anion gap of 21. The patient was admitted to the medical intensive care unit. Intravenous normal saline fluid bolus was given with maintenance intravenous fluid after that. She was kept on nothing per mouth and started on insulin drip with hourly blood glucose check and every four hours serum electrolytes (sodium, potassium, chloride, magnesium, and phosphorus) check.

CT scan of the abdomen showed mild distension of the proximal small bowel and tiny amount of free fluids with wall thickening of distended small bowel loops. She was started on ampicillin/sulbactam after getting blood cultures. Stool sample was sent for analysis and the results were negative leukocytes and negative clostridium difficile toxin. Therefore, we decided to stop the antibiotics at this point and treat her as viral gastroenteritis. Later on, the patient's symptoms have improved and her anion gap closed, so we started her on liquid diet (which she was able to tolerate). She was switched to subcutaneous insulin injections (as insulin glargine at bedtime and sliding scale insulin before each meal) and transferred to medical floor. Her blood cultures did not grow any bacteria, and stool culture was negative. Insulin autoantibodies were negative, c-peptide was lower than normal (0.34 ng/mL), and her glutamic acid decarboxylase autoantibodies were highly positive (>30 U/mL).

After having diabetic education including a dietitian consult in the hospital, the patient was discharged home on subcutaneous insulin treatment with a diagnosis of latent autoimmune diabetes in adult. The patient was encouraged to do regular exercise.

The patient was concerned about her new diagnosis and its possible complications which required counseling about the importance of taking care of her diabetes and how this would help prevent future complications.

The patient was very compliant with the discharge plan and checked her blood sugar before each meal and at bedtime. She followed up in the diabetic clinic on a biweekly basis to adjust her insulin dose. Three months later her hemoglobin A1C was 7.7%. The patient has not experienced any adverse or unanticipated events.

## 3. Discussion

Latent autoimmune diabetes in adult (LADA) is an autoimmune endocrine disorder in which, despite the presence of pancreatic islets antibodies at diagnosis of diabetes, the progression to beta cell failure is slow [1, 2]. Metabolically LADA shares features with both type 1 and type 2 diabetes mellitus. With the former, LADA patients share a severe and progressing defect in beta cell function. With the latter, they share insulin resistance and elevated glucagon levels [9]. Patients are typically diagnosed after 35 years of age and are often misdiagnosed as type 2 diabetes mellitus. Failure to maintain glycemic control with oral-agent combinations and dose adjustment in a lean patient who does not have the usual clinical features of metabolic syndrome should heighten suspicion of LADA [6]. The diagnosis of LADA is currently based on three criteria: (1) adult age at onset of diabetes; (2) the presence of circulating islet autoantibodies (autoantibodies to glutamic acid decarboxylase 65 (GADA 65)/islet cell cytoplasm (ICA)/tyrosine phosphatase like protein (IA-2A)/insulin (IAA)); and (3) lack of a requirement for insulin for at least 6 months after diagnosis [10]. Based on the titer of GADA, LADA has also been subclassified as type 1 and type 2. Patients with higher GADA levels are typed as LADA 1 while patients with lower levels are subclassified as LADA 2 [11]. Patients with LADA do not present with ketoacidosis at the time of diagnosis, owing to the slow progression of beta cell destruction. However, insulin dependency develops at an earlier stage than in type 2 diabetes, and patients in the insulinopenic state are at risk for ketoacidosis [6]. Treatment of LADA, like treatment of type 1 and type 2 diabetes mellitus, should focus on controlling hyperglycemia, preventing complications, and preservation of beta cell function. No treatment guidelines for LADA have been published; therefore patients are mostly treated as affected by type 2 diabetes [3]. Most patients are treated with diet and oral hypoglycemic agents until the secondary failure to treatment with oral hypoglycemic agents results in the need to switch to insulin therapy [12]. Approximately 25% of LADA patients have serological evidence of autoimmune thyroiditis, but there are no reports on the frequency of clinical thyroid disease [10, 13]. Chronic vascular complications, associated with type 1 and type 2 diabetes mellitus, are also present in LADA [10].

In conclusion, diabetic ketoacidosis could be the first manifestation of LADA, although unusual, and should be treated with insulin. Diabetic ketoacidosis can be caused by a number of underlying problems including infections like gastroenteritis in our patient. Poor glycemic control by oral hypoglycemic agent combinations in type 2 diabetic patients who have other autoimmune diseases (like a thyroid disorder) should raise the suspicion of LADA. Although LADA patients tend to have a lower BMI than those with type 2 diabetes mellitus, LADA may affect obese people as was seen in the presented case.
